# Effects of exposure to workplace terrorism on subsequent doctor certified sickness absence, and the modifying role of psychological and social work factors: a combined survey and register study

**DOI:** 10.1186/s12889-020-08465-3

**Published:** 2020-03-30

**Authors:** Mona Berthelsen, Marianne Bang Hansen, Alexander Nissen, Morten Birkeland Nielsen, Stein Knardahl, Trond Heir

**Affiliations:** 1 Norwegian Centre for Violence and Traumatic Stress Studies, Gullhaugveien 1-3, 0484 Oslo, Norway; 2 Oslo University Hospital, Norwegian National Unit for Hearing Impairment and Mental Health, Oslo, Norway; 3 Department of Work Psychology and Physiology, National Institute of Occupational Health, Oslo, Norway

**Keywords:** Psychosocial, Sick-leave, Terror

## Abstract

**Background:**

Studies have shown that terrorist attacks affect the mental and physical health of persons exposed to terrorism. When terror strikes at the workplace where people spend much time, and should feel safe, the health consequences for those affected might be severe. The aim of the study was to determine whether psychological and social work factors moderates effects of exposure to a workplace terrorist attack on subsequent doctor-certified sickness absence.

**Methods:**

The study design combined survey data with register data on sickness absence. Data on exposure to the attack, and psychosocial working conditions were collected by a web-based questionnaire 10 months after the attack. Survey data was linked to registry data on doctor-certified sickness absence over the one-year time period following baseline. The survey response rate was 56% (*n* = 1974), where 80.6% (1591) gave consent to link survey data to data on sickness absence. Exposure to the attack was assessed as “Directly-”, or “Indirectly exposed”. Psychological and social work factors were measured by the General Questionnaire for Psychological and Social factors at Work (QPS_Nordic_). Data were analyzed with negative binominal hurdle regressions.

**Results:**

Direct exposure to the attack increased the odds of becoming sick-listed if *role clarity* was average (OR = 1.50) or high (OR = 2.13), but not if low (OR = 1.17). Direct exposure was associated with higher sickness absence rates if *control over work pace* was low (RR = 1.61). Role conflict, support from co-workers, and -superior showed weaker evidence of moderating effects of exposure on sickness absence.

**Conclusions:**

Exposure to the bomb explosion, as well as psychosocial working conditions affect the risk of employee sickness absence. Psychosocial working conditions seems to moderate effects of exposure to workplace terrorism on subsequent sickness absence. Organizations would benefit from striving for good psychological and social working conditions both as preventions against illness and sickness absence, and as measures in the aftermath of a workplace terrorist attack.

## Background

Terrorist attacks have been shown to affect mental and physical health of exposed individuals including symptoms of anxiety and depression [1], post-traumatic stress disorder [2–4], and musculoskeletal and gastrointestinal pain [5]. Such health problems may affect the workability of workers and contribute to sickness absence [4, 6–9].

Sickness absence in the aftermath of a workplace terrorist attack may be associated with injury or psychological responses to the attack itself, and/or with individual and work-related conditions. To our knowledge, no previous studies have examined sickness absence after a workplace terrorist attack in relation to both disaster exposure, and psychological and social working conditions. We hypothesize that working conditions may moderate effects of exposure on sickness absence in positive and negative ways.

### Mechanisms

Being subjected to terror at work may alter workers’ appraisal of his or her workplace in many ways. The Job Demands-Resources model (JD-R) [10, 11], and The Conservation of Resources (COR) theory [12, 13] may provide a theoretical framework for understanding how stressors may affect worker health, and sickness absence. The JD-R model postulates *job demands* as stressors associated with costs, for example negative health consequences. However, *job resources* may help prevent such negative effects. Job resources are defined as specific physical, psychological, social, and organizational factors of work that may stimulate growth and reduce job demands [10]. For example, if an employee perceive his or her work responsibilities and expectations to be clearly defined, and are provided with autonomy to decide how and when the work should be done, the coping of negative work events, may be bearable compared to if the employee are not allowed to make such adjustments during the workday. Thus, it is reasonable to assume that work factors like autonomy, or support from superior or co-workers may moderate potential adverse health effects of exposure to workplace terrorism on sickness absence. For example, close monitoring and dialogs between employees and supervisor may facilitate recovery, and prevent sickness absence. The COR theory is about loss and gain [12]. The goal is to gain and maintain as many resources as possible (e.g. skills, employment, energy). The theory postulates that stressful conditions, in our case exposure to a workplace terrorist attack, or poor working conditions, will lead to resource losses. Employing resources for coping with such losses is according to Hobfòll (1989) stressful in itself. Thus, it is reasonable to assume that exposure to the terrorist attack combined with role conflicts may put an additional load on the worker, which requires coping resources that in turn may drain energy and induce health problems or sickness absence.

### Workplace terrorism

The body of knowledge of work-related terrorism is limited. A study of employees at Pentagon in the aftermath of the September 11 attacks, 2001, showed that directly exposed workers reported lowered sense of safety at work, especially those who showed posttraumatic stress symptoms or symptoms of depression [14]. Similar results were obtained in a study of Norwegian ministerial employees after the 22th of July, bombing in Oslo in 2011 [15]. Feeling less safe, or experiencing PTSD or symptoms of depression may alter worker’ appraisal of psychological and social working conditions like job demands, leadership or role expectations. Furthermore, traumatized employees may cope with the situation by avoiding the workplace all together, for example through sick leave. A study of Norwegian ministerial employees from pre- to post-disaster showed that perception of leadership generally was stable over time, but workers who experienced posttraumatic stress symptoms perceived their immediate leader to be less supportive [16]. Furthermore, higher levels of social support, and leader support have been shown to be associated with a more rapid decline in worker psychological distress in the aftermath of the 22nd of July Oslo attack [17].

### Workplace terrorism and sickness absence

To our knowledge, only two previous studies have examined associations between exposure to a workplace terrorist attack and subsequent sickness absence. A study of people exposed to the September 11, 2001 attacks in New York showed no statistical significant association between exposure and sickness absence due to psychological or physical symptoms [18]. However, a study of ministerial employees in Norway who were exposed to the 22nd of July, 2011 Oslo bombing showed an increase in sickness absence rates over a two-year period after the attack compared to before the attack [19]. Thus, there is limited evidence of an association between exposure to workplace terrorism and subsequent sickness absence.

Systematic reviews show that working conditions may be important to the mental health of workers in positive and negative ways [20–23]. Furthermore, a systematic review concluded that there is strong evidence that job control and control over working hours may decrease the risk of sickness absence. There are limited evidence for an association between role conflict and role clarity and sickness absence [24]. Another systematic review concluded that there is some evidence that work overload and pressure, lack of control over work, lack of participation in decisions, poor social support and unclear management and work role were associated with increased sickness absence [20]. Based on previous research findings, we elucidate the following specific work factors as potential moderating factors of exposure to the blast on subsequent sickness absence: Role clarity, role conflict, control of decision, control of work pace, support from immediate superior, and support from co-workers.

## Methods

### Design and participants

**Fig. 1:**
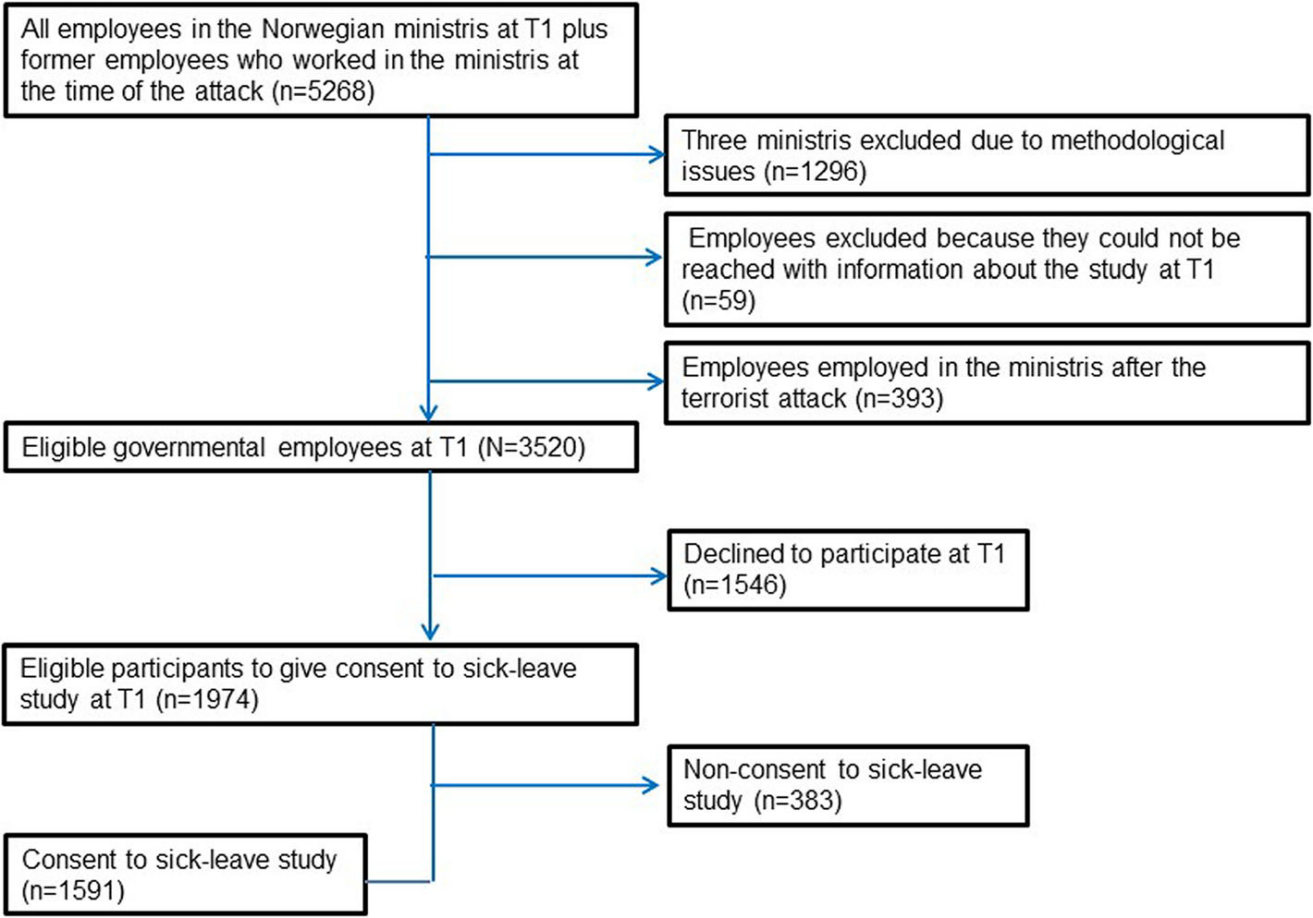
Sample overview and flow of participants

The current study is based on survey-data of employees from the governmental ministries in Norway, and doctor-certified sickness absence data. The survey was conducted 10 months (May to August, 2012) after the 22nd of July, 2011 bomb attack in Oslo (Fig. 1). Doctor certified sickness absence data was obtained from Statistics Norway and the Norwegian Labor and Welfare Administration. A total of 1651 workers gave their consent to link survey-data to data of doctor certified sickness absence (Fig. 1).

### Organizational context

Eight people were killed in the attack, and about 200 more were wounded. The explosion caused massive damage to the infrastructure in the government quarter. Thirteen of the 14 participating ministries were located near the epicenter of the bomb explosion. Because of extensive damage to facilities, several ministries had to relocate immediately after the terrorist attack.

### Procedures

The data collection was carried out by the National Institute of Occupational Health in Norway using a web-based questionnaire. All employees were mailed a letter with information about the aims of the survey, the data collection procedures, and a personal log-in code to the web-questionnaire. The National Institute of Occupational health has a license from the Norwegian Data Inspectorate to gather and store health information by the web-based questionnaire system. The participants were informed that responses would be stored and handled according to strict rules to secure, and maintain responder confidentiality. The respondents gave their consent to participation by replying to the survey. The questionnaire was accessible from any location with an internet connection. Participants were allowed to log in an unlimited number of times to complete their answers. The survey has been approved by the Regional Committee for Medical and Health Research Ethics, South East, Norway.

### Instruments

#### Exposure to the bomb attack

Exposure to the bomb attack was measured by the following item “Where were you on the 22. July 2011 when the bomb exploded?”, The response alternatives were (1) In the Government district, (2) Downtown Oslo, but not in the Government district, (3) In Oslo, but not downtown, (4) In Norway, but not in Oslo, and (5) Abroad. The response categories were dichotomized into (1) “direct exposure” comprising response alternative one, and (0) “indirect exposure” comprising response alternatives two to five.

#### Psychological and social work exposures

*Role clarity*, *role conflict*, *control of decision*, *control of work pace*, *support from superior*, and *support from coworkers* were measured by the General Questionnaire for Psychological and Social factors at Work, QPS_Nordic_ [25]. Role clarity comprised three items, e.g. “Do you know exactly what is expected of you at work?” (Cronbachs alpha = .80). Role conflict comprised three items, e.g. “Do you receive incompatible requests from two or more people?” (Cronbachs alpha = .66). Control of decision comprised four items, e.g. “If there are alternative methods for doing your work, can you choose which method to use?” (Cronbachs alpha = .75). Control of work pacing comprised four items, e.g. “Can you set your own work pace?” (Cronbachs alpha = .80). Support from superior comprised three items, e.g. “If needed, can you get support and help with your work from your immediate superior?” (Cronbachs alpha = .87). Support from coworkers comprised two items, e.g. “If needed, are your coworkers willing to listen to your work-related problems?” (Cronbachs alpha = .81). The response categories for all items referred to the frequency of occurrence and ranged from 1 (Very seldom or never), 2 (Rather seldom), 3 (Sometimes), 4 (Rather often), and 5 (Very often or always). Missing response to one of the items comprising each scale was allowed.

#### Sickness absence

Data on sick-leave was obtained from registry data on doctor-certified sickness absence from the Norwegian Labour and Welfare Administration registry, and data on employment from Statistics Norway. The registry of employment contains the number of expected workdays for each quarter for a given employee based on the employee’s labor contract. Vacation, weekends and public holidays are not included as workdays. Thus, there are approximately 226 expected workdays a year for a person in full-time employment. The registry on sick-leave contains the number of days a given employee was absent from work per quarter due to doctor-certified sick-leave, adjusted for whether the sick-leave is graded or if the employee works full-, or part-time. For example, if an employee who works 60% (three days a week) gets two weeks of 50% sick-leave in a quarter, the employee will have three registered days of sickness absence for that quarter. The outcome variable comprises the total of sickness absence days for the one-year time-period from baseline (T1) and one year ahead (second, third and fourth quarter 2012, and first quarter 2013). Thus, the outcome variable comprises standardized sickness absence days of full-time employment for each participant.

### Statistical analyses

#### Non-response analyses

Potential confounding effects of non-response were estimated by multivariate binary logistic regression analysis under “Generalized linear models” (GZLMs) in SPSS version 25. The odds of being a responder at baseline were estimated based on sex (male = 0 and female = 1) and age of eligible participants. Male was chosen as reference category. Age was categorized into the following age groups: 1) 19–29 years (*n* = 254), 2) 30–39 years (*n* = 840), 3) 40–49 (*n* = 997), 4) 50–59 (*n* = 755), and 5) 60–70 years (*n* = 384). Age group 1 was chosen as reference category.

#### Attrition analyses

Potential confounding effects of non-consent to linking survey data with registry data on sickness absence were estimated by binary logistic regression analyses. Effects of sex, age, education, role conflict, role clarity, control over decision, control over work pace, support from immediate superior, and support from co-workers were analyzed separately. Sex, age and education were entered as categorical variables, and the remaining as continuous variables.

#### Hurdle regressions

The outcome variable sickness absence days was expressed as count data, were the most frequent score was zero, no sickness absence days. The outcome variable was also characterized with variance larger than the mean (overdispersion) [26]. Thus, the present study employed negative binomial hurdle models [27] using the package *pscl* in R to estimate sickness absence. Hurdle regressions are to-part models, were a binomial logistic regression model are used to estimate the odds of having at least one day of sickness absence, expressed as odds ratios. The second part of hurdle regression uses a negative binomial regression model to estimate the mean number of days with sick-leave among those who have at least one day of sickness absence, expressed as rate ratios. All parameters in hurdle are estimated by maximum likelihood using *optim*. Maximum likelihood is a method of nonlinear model fitting that applies even if the errors are not normal. The method finds the parameter values which maximize the log likelihood, or equivalently which minimize the negative log-likelihood.

Effects of exposure to workplace terrorism, and effects of psychological and social work factors were modelled in six separate hurdle regression analyses, by adding one work factor to the exposure variable in each model. Interaction effects were modelled by estimating odds ratio and rate ratio for the exposure variable by each of the moderator variables in separate models. Each work factor (included as a continuous variable in the model) was centered on its mean, representing “average” value for the work factor in question. Then, odds ratios and rate rations for “low”, “average”, and “high” values for each work factor were estimated. These were as follows: Role clarity, “Low” score = 3.5, and “high” score = 4.5. Role conflict, “low” score = 2.0, and “high” score = 3.0. Control over decision, “low” score = 2.7, and “high” score = 3.8. Control over work pace, “low” score = 3.3, and “high” score = 4.2. Support from leader, “low” score=3.4, and “high” score = 4.4. Support from co-workers, “low” score = 3.5, and “high” score = 4.5.

Analyses were conducted in R (The R Foundation for Statistical Computing, Vienna, Austria, 2018), with the R packages pscl for hurdle regression (R Development Core Team (2017).

## Results

### Non-response analysis with regard to the survey design

The study population comprised all eligible employees in 14 out of 17 Norwegian ministries on the 22 of July 2011 (*n* = 3520). A total of 1974 employees (56.08%) completed at least parts of the questionnaire, whereas 1546 declined to participate (Fig. 1). Binary logistic regression analysis with sex and age as predictors showed that females had higher odds of responding at baseline compared to males (OR = 1.37, *p* < 0.001). Age was not associated with being a responder at baseline (ORs ranging from 0.95 to 1.15, *p*-values ranging from 0.329 to 0.711).

### Attrition analyses with regard to consent to link survey data to registry data on sickness absence

Of the 1974 participants who responded at baseline, 1591 (80.6%) participants gave consent to linking survey data to registry data on sickness absence (Fig. 1). Binary logistic regression analyses revealed that females had lower odds of giving consent compared to males (OR = 0.75, *p* = 0.025). Age was not associated with giving consent (ORs ranging from 0.87 to 1.13, *p*-values ranging from 0.557 to 0.857). Having less than 13 years of education (OR = 0.71, *p* = 0.017), and between 13 to 16 years of education (OR = 0.57, *p* = 0.002) decreased the odds of giving consent compared to having more than 16 years of education. Role clarity (OR = 1.08, *p* = 0.410) role conflict (OR = 1.18, *p* = 0.280), and support from co-workers (OR = 1.18, *p* = 0.072) were not associated with giving consent. Control over decision (OR = 1.47, *p* < 0.001), control over work pace (OR = 1.27, *p* = 0.011), and support from immediate superior (OR = 1.22, p = 0.011) increased the odds of giving consent.

### Sample characteristics

The final sample comprised slightly more females (56.3%, *n* = 895) than males (43.7%, *n* = 696). The average age was 45.5 years (SD = 11.02). The education level was high with 66.7% (*n* = 1061) of participants having more than 16 years of education, 23.4% (*n* = 373) had 13 to 16 years of education, and 9.9% (*n* = 157) less than 13 years of education. One hundred and seventy five participants (11%) of the sample reported being present at work in the Governmental district when the bomb went off.

### Bivariate associations between predictor variables and outcome

Higher education, higher control over decision, control over work pace, support from superior, and support from co-workers were associated with less sickness absence days (Table 1). Female sex, exposure to the bomb explosion, and higher role conflict were associated with more sickness absence days. The correlations between predictor variables were small to moderate, except the correlation between support from superior and support from co-workers which may be considered high (rho = .63). However, this does not pose a risk of collinearity in the regression models as each work factor was analyzed in separate regression models.

**Table 1:** Spearman’s correlation between age, sex, exposure to the bomb explosion, psychological and social work factors and sickness absence days

	1	2	3	4	5	6	7	8	9	10	11	12
1. Age												
2. Education	−.21^**^											
3. Sex	−.09^**^	−.08^**^										
4. Exposure	-.01^ns^	-.01^ns^	.03^ns^									
5. Role clarity	.16^**^	−.20^**^	.01^ns^	-.03^ns^								
6. Role conflict	−.12^**^	.06^*^	.02^ns^	.05^*^	−.34^**^							
7. Control decision	.10^**^	.17^**^	−.12^**^	−.08^**^	.24^**^	−.21^**^						
8. Control work pace	-.01^ns^	.13^**^	-.01^ns^	−.10^**^	-.00^ns^	−.19^**^	.43^**^					
9. Support superior	−.03 ^ns^	.01^ns^	-.04^ns^	−.08^**^	.41^**^	−.39^**^	.36^**^	.24^**^				
10. Support co-worker	−.11^**^	.02^ns^	.07^**^	−.09^**^	.30^**^	−.31^**^	.30^**^	.27^*^	.63^**^			
11. Sickness absence days	.04^ns^	−.16^**^	.20^**^	.07^**^	.01^ns^	.05^*^	−.17^**^	−.09^**^	−.10^**^	−.07^**^		
12. Sickness absence days one year prior to 22. July	.04^ns^	−.12^**^	.18^**^	-.02^ns^	-.04^ns^	.05^*^	−.13^**^	−.07^**^	−.10^**^	-.05^ns^	.30^**^	
Mean	45.50				3.92	2.52	3.33	3.86	3.94	4.06	9.54	7.61
SD	11.02				0.73	0.70	0.67	0.71	0.86	0.76	27.46	23.81
Min-max	20–70				1–5	1–5	1–5	1–5	1–5	1–5	0–250	0–243

^*^p < .05

^**^p < .01

^ns^ = not significant

### Hurdle regressions

The odds of becoming sick-listed and sickness absence rates were higher among females compared to males. Older age was associated with higher sickness absence rates (duration of sickness absence among those with sick-leave). Employees with less than 13 years of education, and employees with 13 to 16 years of education showed higher odds of becoming sick-listed compared to employees with more than 16 years of education.

Direct exposure to the bomb explosion was associated with higher odds of sickness absence, as well as higher sickness absence rates compared to indirectly exposed employees (Table 2). Higher levels of role conflict were associated with higher odds of sickness absence, independently of exposure. Higher levels of control over decision, support from superior, and support from co-worker were associated with lower odds of becoming sick listed independently of exposure, (Table 2). Support from superior was also associated with lower sickness absence rates.

**Table 2:** Effects of exposure to the bomb explosion, and work factors on subsequent sickness absence

	Binomial logistic regression^a^			Negative binomial regression^a^		
	OR	95%CI	*P*-value	RR	95%CI	*P*-value
Sex female	2.26	[1.78–2.87]	< 0.001	1.33	[1.02–1.73]	0.034
Age	1.05	[0.94–1.17]	0.131	1.13	[1.00–1.26]	0.045
Education:						
More than 16 years	ref.					
13–16 years	1.44	[1.10–1.88]	0.008	0.98	[0.733–1.32]	0.915
Less than 13 years	3.36	[2.34–4.84]	< 0.001	1.07	[0.76–1.51]	0.677
Indirectly exposed	ref.					
Directly Exposed	1.47	[1.04–2.07]	0.028	1.53	[1.07–2.19]	0.021
Role clarity	0.89	[0.76–1.04]	0.131	0.98	[0.83–1.16]	0.825
Role conflict	1.26	[1.07–1.48]	0.007	1.15	[0.97–1.35]	0.116
Control over decision	0.65	[0.54–0.77]	< 0.001	0.88	[0.74–1.04]	0.124
Control over work pace	0.86	[0.74–1.01]	0.071	0.99	[0.84–1.16]	0.862
Support from superior	0.80	[0.70–0.91]	< 0.001	0.85	[0.75–0.96]	0.011
Support from co-worker	0.77	[0.66–0.90]	< 0.001	0.92	[0.79–1.07]	0.268

OR odds ratio

RR rate ratio

^a^ All work factors were analyzed in separate models adjusted for sex, age, education, and exposure to the bomb explosion

There was evidence of a moderating role of work factors on the effects of exposure to the bomb explosion and subsequent sickness absence (Table 3). Direct exposure to the attack increased the odds of becoming sick-listed when *role clarity* was average or high, but not when role clarity was low. Exposure to the attack was associated with higher sickness absence rates if *control over work pace* was low. Moderating effects of role conflict, control of decision, support from immediate superior, and support from co-workers did not reach statistical significance [28] (Table 3).

**Table 3:** Moderating effects of work factors on exposure to the bomb explosion and subsequent sickness absence

	Binomial logistic regression^a^			Negative binomial regression^a^		
	OR	95% CI	*P*-value	RR	95% CI	*P*-value
Exp*role clarity			0.012			0.183
Effect of exposure if role clarity is:						
Low	1.17	[0.79–1.75]	0.433	1.69	[1.11–2.57]	0.014
Average	1.50	[1.06–2.13]	0.023	1.49	[1.05–2.11]	0.027
High	2.13	[1.37–3.31]	< 0.001	1.24	[0.82–1.87]	0.313
Exp*role conflict	0.65	[0.40–1.05]	0.078	1.51	[0.92–2.48]	0.102
Effect of exposure if role conflict is:						
Low	1.90	[1.21–2.98]	0.005	1.11	[0.90–1.29]	0.402
Average	1.52	[1.07–2.15]	0.018	1.37	[0.97–1.93]	0.078
High	1.22	[0.82–1.82]	0.319	1.67	[1.11–2.51]	0.013
Exp*ctrl decision	1.42	[0.87–2.32]	0.165	0.86	[0.53–1.41]	0.558
Effect of exposure if control of decision is:						
Low	1.21	[0.80–1.83]	0.374	1.56	[1.05–2.32]	0.029
Average	1.49	[1.05–2.12]	0.025	1.43	[1.00–2.05]	0.053
High	1.77	[1.12–2.82]	0.016	1.33	[0.82–2.16]	0.253
Exp*ctrl work pace			0.511			0.022
Effect of exposure if control of work pace is:						
Low	1.52	[1.04–2.22]	0.032	1.61	[1.11–2.34]	0.012
Average	1.40	[0.98–2.00]	0.068	1.14	[0.77–1.67]	0.520
High	1.32	[0.86–2.03]	0.204	0.90	[0.54–1.49]	0.681
Exp*support superior	1.44	[0.98–2.12]	0.062	0.74	[0.52–1.07]	0.108
Effect of exposure if support from immediate superior is:						
Low	1.25	[0.87–1.81]	0.241	1.53	[1.05–2.21]	0.025
Average	1.51	[1.07–2.14]	0.020	1.31	[0.93–1.84]	0.128
High	1.80	[1.18–2.74]	0.006	1.13	[0.76–1.70]	0.543
Exp*Support co-workers	1.40	[0.90–2.18]	0.140	0.69	[0.46–1.03]	0.071
Effect of exposure if support from co-workers is:						
Low	1.26	[0.85–1.85]	0.250	1.60	[1.09–2.35]	0.017
Average	1.51	[1.06–2.14]	0.022	1.31	[0.92–1.85]	0.136
High	1.76	[1.13–2.72]	0.012	1.10	[0.72–1.68]	0.655

OR odds ratio

RR rate ratio

^a^ All work factors were analyzed in separate models adjusted for sex, age, and education

## Discussion

The present study showed that psychological and social work factors appear to be important both for the odds of becoming sick-listed, and for the duration of the sickness absence period in the aftermath of a workplace terrorist attack. The present study also showed a complex moderating relationship between exposure to the terrorist attack, psychological and social work factors and effects on subsequent sickness absence.

Directly exposure to the terrorist attack was associated both with increased odds of becoming sick-listed and increased duration of the sickness absence period (also see Hansen et al., under review). A valid basis for comparison of results is currently lacking. A study by Osinubi and collegues (2008) did not find and association between exposure to the September 11 attack and subsequent sickness absence due to psychological or physical diagnoses. However, the current study did not analyze diagnosis-specific sickness absence. Furthermore, sickness absence in Norway may not be comparable to sickness absence in the USA because of highly different rules of sickness absence in the two countries. In Norway, loss of income due to sickness absence is compensated by the Norwegian National Insurance for maximum six times the Norwegian national insurance schemes’ basic amount of 93,634 NOK. Hence, workers do not suffer extensive economic losses during the first year absent from work. Income above this amount will not be compensated.

Control of decision and leader- and co-worker support were associated with decreased odds of becoming sick-listed. Thus, our findings are in line with previous findings in the field [20, 24]. Support from superior was associated with decreased duration of sickness absence. Hence, job control and support may act as protective factors against becoming sick-listed in the first place, and as protective of long-term sickness absence. Role conflict, on the other hand, appeared to increase the odds of becoming sick-listed, and prolong the duration of sickness absence. These findings support previous findings in the field [20, 24].

Effects of exposure to a workplace terrorist attack on subsequent sickness absence appeared to interact in a complex manner with psychological and social working conditions. Exposure to the blast appeared to increase the odds of sickness absence when role clarity was average or high, but not when role clarity was low. Role clarity is usually considered as protective of sickness absence [25], and this notion have gained some support empirically [20, 24]. There is no obvious reason for why exposure should increase the odds of sickness absence with higher levels of role clarity. However, a speculation may be that being highly aware of one’s responsibilities and expectations actually may act as a work load when coping with psychological reactions following a workplace terrorist attack, especially when work ability may be reduced. Another explanation may be that the initial level of sickness absence may be higher among workers with low role clarity than workers with high role clarity. Consequently, the effect of exposure on sickness absence may not be equally visible with different levels of role clarity as the effect is overshadowed by high initial levels of sickness absence in this group [29]. The current findings partly support previous findings of associations between role clarity and sickness absence [20, 24].

As expected, exposure to the bomb explosion appeared to increase the rates of sickness absence when control of work pace was low, but not when average or high. Hence, facilitating worker autonomy may protect against long-term sickness absence in the aftermath of terrorism or other stressful events at the workplace.

Potential modifying roles of role conflict (*p* = 0.078), support from superior (*p* = 0.062), and support from co-workers (*p* = 0.071) on sickness absence were marginally not statistically significant, and might be worth wile further investigation in future studies as well. We believe the current results gives an insight into the complexity in studying and interpret moderation when it comes to the issue of type I and type II errors [28], as well as studying moderation on multiplicative scales [29]. Role conflict is generally considered to be a risk factor for ill-health [25]. The current findings support this notion (Table 2) and are in line with previous studies [24, 30, 31]. However, effects of exposure to a workplace terrorist attack on subsequent sickness absence seems to be more pronounced with lower levels of role conflict than higher. As discussed earlier, this may be explained by initially higher levels of sickness absence among those who experience high role conflict overshadowing the effect of exposure.

Support from superior is generally considered to be a protective factor of ill-health [25]. However, the empirical evidence is mixed with regard to sickness absence [24]. The current study showed that the odds of becoming sick-listed decreased with increasing levels of support from superior. As with our previous findings, the effect of exposure on sickness absence seems to be more pronounced with higher levels of leader-support, which might be an indication of leader-support initially acting as a protective factor of sickness absence. Hence, facilitating leader-support might be important for reducing the risk of sickness absence.

### Strengths and limitations of the study

Strengths of the current study were its fairly high sample size, the prospective study design, and the combination of different data sources. By combining survey data of exposures (predictor variables) and registry data on sickness absence (outcomes), the present study minimizes the risk of observing spurious associations that could be attributed to common method bias [32]. However, as the predictor variables of the present study were assessed by self-report measures, we cannot rule out the potential problems reporting bias associated with such measures poses [32]. The QPS_Nordic_ [25] instrument used in the current study to assess job control, role clarity, role conflict, support from immediate superior, and support from co-workers should be relatively insensitive to respondents’ personality dispositions or emotions. Negative/positive connotations in response scales, using verbal labels for all response categories, and reversing some of the items were measures employed in construction of the items to reduce the risk of reporting bias. The respondents were asked how often a situation occurs instead of degrees of agreement or satisfaction [25]. The cronbach’s alpha coefficient for role conflict was .66, thus the precision of the measurement may have been weakened in the current study.

The response rate at survey-baseline was 56% and thus above the estimated average for organizational surveys [33]. In terms of selection bias, we know that females were more likely to respond at baseline compared to males. A low response rate may pose a threat to internal validity through self-selection mechanisms if participating is a common effect of both exposure and outcome [32]. Of the 1974 workers who responded at baseline, 83.6% gave their consent to link survey data to data on sickness absence, which may be considered highly acceptable in terms of representativeness. Still, lower education and being female was associated with non-consent. Although, exposure to the terrorist attack may have been a focus of motivation to participate in the survey, adverse working conditions and sickness absence were likely not.

There is a chance that workers who became sick-listed in the time period from the terrorist attack to baseline never was reached when conducting the survey 10 months after the attack. Furthermore, three out of seventeen ministries declined to participate in the survey, and may pose a risk of selection bias.

The current data on sickness absence includes doctor-certified sickness absence only, not self-reported sickness absence. In Norway, employees are allowed to be absent from work for up to three consecutive days, four times a year without a sick-leave note from a doctor. If the organization of employment is a part of “The agreement on inclusive working conditions”, a employee is allowed to be absent from work for up to eight consecutive days, for a maximum of 21 days a year without a sick-leave note from a doctor. It is likely that the overall absenteeism from work due to health complaints was even higher than the current study’s estimate.

A debated issue in research on sickness absence is whether or not one should include previous sickness absence in analyses of future sickness absence [34]. At baseline, there is already an association between exposure to the bomb explosion, working conditions and sickness absence (10 months after the attack). Also, employees may have become sick-listed in the period from immediately after the blast to baseline. We believe the likelihood of a substantial increase in sickness absence from 10 to 22 months after the attack is low because the level of sickness absence was already high for some employees at baseline. Adjustment for previous sickness absence in the regression models rules out the variance in sickness absence at baseline explained by the predictors. Hence, we may risk underestimation of the true effect of exposure to the blast and working conditions on sickness absence.

The current study aimed at elucidating potential moderating effects of working conditions on effects of exposure to the bomb explosion on sickness absence. However, as Table 1 suggests, exposure to the blast seemed to affect perceptions of working conditions at baseline. We cannot rule out the possibility that working conditions may mediate effects of exposure to the blast on subsequent sickness absence.

## Conclusions

The present study showed that exposure to the bomb explosion, and role conflict were risk factors for subsequent sickness absence. Control over decision, control over work pacing, and support from superior and co-workers were found to be protective factors of sickness absence. We found support for moderating effects of role clarity and control over work pace, and weaker evidence of moderating effects of role conflict, support from co-workers, and support from leader. The patterns of interactions are complex, and conclusions should be drawn with caution. Directly and indirectly exposed workers may benefit from good working conditions in order to lower the risk of sickness absence and shorten sickness absence periods. Organizations in general, and organizations exposed to workplace terrorism, would benefit from striving for good psychological and social working conditions both as preventions against illness and sickness absence, and as measures in the aftermath of a workplace terrorist attack.

## Data Availability

The datasets used and/or analyzed during the current study are available from the corresponding author on reasonable request. The scales on psychological and social work factors employed in the current study is published in Dallner, M., et al., *Validation of the General Nordic Questionnaire (QPSNordic) for psychological and social factors at work*. NORD (Nordisk ministerråd: trykt utg.). Vol. 2000:12. 2000, København: Nordisk Ministerråd.

## Abbreviations

QPS_Nordic_: The General Questionnaire for Psychological and Social factors at Work
OR: Odds ratio
RR: Rate ratio
JD-R: The Job Demands-Resources model
COR: The Conservation of Resources theory
